# Venetoclax with decitabine or azacitidine in the first‐line treatment of acute myeloid leukemia

**DOI:** 10.1002/jha2.663

**Published:** 2023-02-24

**Authors:** Ian M. Bouligny, Graeme Murray, Michael Doyel, Tilak Patel, Josh Boron, Valerie Tran, Juhi Gor, Yiwei Hang, Yanal Alnimer, Kyle Zacholski, Chad Venn, Nolan A. Wages, Steven Grant, Keri R. Maher

**Affiliations:** ^1^ Division of Hematology and Oncology Department of Internal Medicine Virginia Commonwealth University Massey Cancer Center Richmond Virginia USA; ^2^ Virginia Commonwealth University School of Medicine Richmond Virginia USA; ^3^ Department of Internal Medicine Virginia Commonwealth University Medical Center Richmond Virginia USA; ^4^ Department of Pharmacy Virginia Commonwealth University Medical Center Richmond Virginia USA; ^5^ Department of Biostatistics Virginia Commonwealth University School of Medicine Richmond Virginia USA

**Keywords:** acute leukemia, AML, BCL2, chemotherapy, clinical research, malignant hematology

## Abstract

Treatment paradigms for acute myeloid leukemia (AML) have evolved at a rapid pace in recent years. The combination of venetoclax with a hypomethylating agent prolonged survival in clinical trials when compared to hypomethylating agent monotherapy. However, little is known about the performance of venetoclax‐based regimens outside of clinical trials, given conflicting safety and efficacy data. Even less is known about the impact of the hypomethylating agent backbone. In this study, we demonstrate that decitabine‐venetoclax is associated with a significantly higher rate of grade three or higher thrombocytopenia, but lower rates of lymphocytopenia compared to azacitidine‐venetoclax. There was no difference in response or survival across ELN 2017 cytogenetic risk categories in the overall cohort. Significantly more patients succumb to relapsed or refractory disease than death from any other cause. We demonstrated that a Charlson comorbidity index score threshold of seven identifies exceptionally high‐risk patients, providing evidence for clinical use to reduce the risk of early treatment‐related mortality. Lastly, we provide evidence that measurable residual disease negativity and an *IDH* mutation predict a significant survival benefit outside clinical trials. Taken together, these data illuminate the real‐world performance of venetoclax and decitabine or azacitidine in the treatment of AML.

## 1 INTRODUCTION

Acute myeloid leukemia is a heterogenous bone marrow neoplasm that reflects an arrest in the development of hematopoietic precursor cells [1]. AML primarily occurs at a median age of 68 years and is associated with decreased survival with increasing age [2, 3]. Intensive therapies are commonly followed by consolidation and allogeneic stem cell transplant (SCT) — a treatment paradigm for which many older adults are ineligible. Therefore, there is increasing interest in augmenting existing treatment strategies in elderly patients, particularly with small‐molecule inhibitors of B‐cell lymphoma 2 (BCL‐2).

**FIGURE 1:**
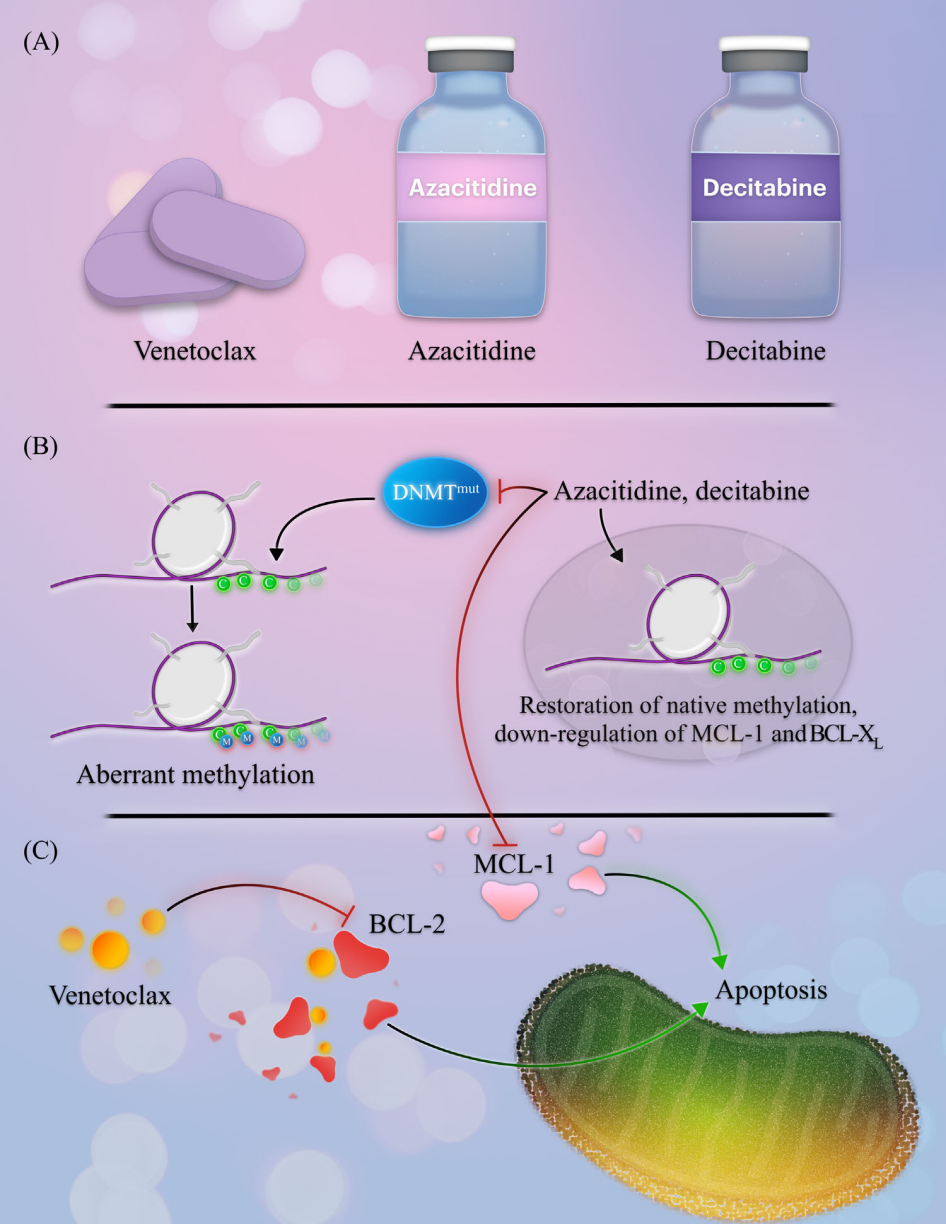
Overview of venetoclax, azacitidine, and decitabine. (A) Venetoclax is orally administered concurrently with azacitidine or decitabine. Azacitidine and decitabine are administered either intravenously or subcutaneously. (B) Hypomethylating agents abrogate aberrant methylation patterns in acute myeloid leukemia (AML), reversing malignant methylation signatures. They also downregulate the pro‐survival proteins myeloid cell leukemia‐1 (MCL‐1) and B‐cell lymphoma extra‐large (BCL‐X_L_). (C) Venetoclax inhibits BCL‐2, freeing pro‐apoptotic proteins that potentiate myeloid blast apoptosis.

BCL‐2 is an apoptotic‐regulating protein that is frequently overexpressed in AML [4, 5]. BCL‐2 binds and inhibits primary effectors of apoptosis: BAX and BAK. Small‐molecule inhibitors of BCL‐2 free BAX and BAK, eradicating myeloid blasts and leukemic stem cells [4, 6]. Venetoclax is an orally available, small‐molecule BCL‐2 homology domain 3 (BH3) mimetic that inhibits BCL‐2 [6]. However, venetoclax has limited efficacy as monotherapy in AML, partially due to primary and adaptive resistance [7]. Important mechanisms of venetoclax resistance include the upregulation of the pro‐survival proteins myeloid cell leukemia‐1 (MCL‐1) or BCL‐X_L_ [8, 9]. The hypomethylating agents, decitabine and azacitidine, downregulate MCL‐1 and BCL‐X_L_ and reduce venetoclax resistance, as shown in Figure 1 [9, 10, 11]. In addition, the hypomethylating agents inhibit DNA methyltransferases, induce tumor suppressor genes, and incorporate into either DNA in the case of decitabine or RNA with azacitidine [12, 13, 14]. These observations provided the rationale for clinical trials evaluating the combination of hypomethylating agents and venetoclax.

In the VIALE‐A trial, venetoclax and azacitidine demonstrated significantly longer overall survival at 14.7 months compared to 9.6 months with azacitidine alone [10]. The use of venetoclax with a hypomethylating agent has since become routine in the treatment of AML. Despite this, outcomes outside clinical trials and analyzed with respect to the selection of the hypomethylating agent backbone remain unclear. Large population‐based studies investigating azacitidine versus decitabine as monotherapy showed a small survival benefit for decitabine‐treated patients. However, the significance of the survival benefit disappeared after analyzing patients that completed the intended schedule of chemotherapy [15].

The efficacy of different hypomethylating agent backbones in combination with venetoclax requires clarification. Retrospective analyses are conflicting, with no definitive differences in response rates between azacitidine‐venetoclax and decitabine‐venetoclax in the first‐line setting [16]. In one retrospective study, the median overall survival significantly favored the azacitidine‐venetoclax group at 12.3 months compared to 2.8 months with decitabine‐venetoclax [17]. In contrast, other retrospective analyses showed a non‐significant survival benefit favoring decitabine‐venetoclax over azacitidine‐venetoclax [18]. Moreover, treatment‐related adverse events and results stratified by ELN cytogenetic risk outside clinical trials are lacking.

## 2 METHODS

### 2.1 Objectives

The two primary objectives of this study were to retrospectively determine the composite complete remission rate and overall survival of patients with newly diagnosed AML treated with venetoclax with decitabine or azacitidine. The secondary objectives were to determine patient‐ and disease‐related predictors of survival, assess the survival in patients that achieved a response negative for measurable residual disease (MRD), and characterize toxicities associated with venetoclax and decitabine or azacitidine.

### 2.2 Patient eligibility

The Institutional Review Board of Virginia Commonwealth University Medical Center approved this single‐center, retrospective protocol involving 74 patients analyzed across 165 treatment phases. The eligibility criteria included all patients aged 18 years or older with newly diagnosed AML that received at least one dose of venetoclax with decitabine or azacitidine from January 2018 to January 2022. Patients were excluded if death occurred before the first dose of disease‐directed therapy or if treatment records were unavailable for retrospective analysis.

### 2.3 Treatment regimens

Patients were treated with venetoclax starting on day one of treatment and continuing until the end of the 28‐day cycle or shorter duration, adjusted for toxicity or drug‐drug interactions. Venetoclax was administered in 28‐day cycles with decitabine 20 mg/m^2^ in 5‐ or 10‐day courses or azacitidine 75 mg/m^2^ in 5‐ or 7‐day courses. Venetoclax and decitabine or azacitidine were then administered as maintenance in 28‐day cycles until intolerability, disease progression, or death, with cycle delays allowed for adverse events or count recovery.

### 2.4 Data collection and entry

We designed a REDCap instrument to retrospectively capture patient data [19]. The instrument was programmed to include cytogenetic and molecular profiles, response, and toxicity for each phase of treatment, including induction, maintenance, and relapse. Built‐in score calculators and survival computation were programmed into the instrument during development to standardize data entry among investigators and minimize the likelihood of analytical errors.

The lead investigator reviewed the data set at two pre‐specified time points and cross‐checked entries for accuracy with the electronic medical record (Cerner Millennium and Epic). A minimum of two investigators standardized and cross‐checked response and toxicity grading. Data discrepancies were resolved following a review by the lead investigator.

### 2.5 Safety analysis

Toxicities were graded using the Common Terminology Criteria for Adverse Events (CTCAE) version 5.0 [20, 21]. Treatment‐related adverse events were included if they occurred between the first dose and 28 days following treatment discontinuation. Quantitative toxicities were graded and recorded throughout each patient's treatment phase, excluding electrolyte aberrations. In instances where complete records were unavailable, toxicities were marked as unavailable for the phase of treatment to reduce bias.

### 2.6 Cytogenetic, molecular, and measurable residual disease analyses

AML was defined using the fourth edition World Health Organization criteria, with a minimum of one bone marrow biopsy demonstrating at least 20% or greater myeloblasts [22]. The cytogenetic risk was defined as recommended by the European LeukemiaNet 2017 guidelines [23]. PCR assays obtained at diagnosis had a sensitivity of 10^−4^ for *NPM1* and 10^−2^ for *CEBPA*, *FLT3*‐ITD, and *FLT3*‐TKD. Next‐generation sequencing (NGS) was performed using an in‐house NGS assay with a sensitivity of 2.7 × 10^−2^.

MRD negativity was defined using an assay at a minimum sensitivity threshold of 10^−3^, including PCR‐based MRD assays and multiparameter flow cytometry (MFC; University of Washington Medical Center). Mutations frequently associated with clonal hematopoiesis, including *DNMT3A*, *TET2*, and *ASXL1*, were not considered MRD if detected on a remission NGS assay [24]. Similarly, germline mutations, such as *DDX41*, *GATA2*, and *TP53*, were excluded as MRD [24]. MRD‐negative results with suboptimal sample quality, as indicated in the result report, were excluded from MRD analysis.

### 2.7 Response assessment

Response assessments were performed in accordance with the modified International Working Group response criteria for AML [25]. Complete remission (CR) was defined as an absolute neutrophil count (ANC) of greater than 1000 cells/mm^3^, a platelet count of greater than 100,000 cells/mm^3^, transfusion independence, and a bone marrow biopsy with less than 5% blasts. CR with incomplete hematologic recovery (CRi) was defined as all the criteria for CR except for neutropenia (ANC ≤ 1000 cells/mm^3^) or thrombocytopenia (platelets ≤ 100,000 cells/mm^3^). CR with partial hematologic recovery (CRh) was defined as all the criteria for CR except for lower ANC (>500 cells/mm^3^) and platelet (>50,000 cells/mm^3^) thresholds. Progressive disease was defined as outlined by the European LeukemiaNet guidelines [23]. Composite complete remission (CRc) included patients that achieved CR, CRi, or CRh.

### 2.8 Statistical analysis

Patients treated between January 1, 2018, and January 1, 2022, were included in the study. The clinical data cutoff date was August 1, 2022, and patients alive at that time were censored. Means between groups were compared using the nonparametric Mann‐Whitney test. Remission rates were reported with 95% confidence intervals using the Wilson method and compared between groups using Fisher's exact test. The overall survival was estimated for each cohort using the Kaplan‐Meier method and compared using the log‐rank test. The hazard ratio was estimated using the Cox proportional hazards model. Cox proportional hazards assumptions were assessed using graphical methods and tested using Schoenfeld residual analysis with a level of significance of 0.01. All reported *p*‐values were two‐sided, with statistical significance evaluated at the 0.05 alpha level. Data analysis was performed with GraphPad Prism 9.4.1 for Macintosh.

## 3 RESULTS

### 3.1 Demographics and baseline characteristics

We identified 74 patients with newly diagnosed AML treated with venetoclax and decitabine or azacitidine. The median age at diagnosis was 73 years (range, 26–85 years). Fourteen (19.7%) were ELN 2017 favorable risk, 15 (21.1%) were intermediate risk, and 42 (59.2%) were adverse risk. We evaluated patient fitness using the Charlson comorbidity index (CCI) score and Eastern Cooperative Oncology Group (ECOG) score captured at diagnosis. The median CCI score was 6 (range, 3–12), and the median ECOG score was 2 (range, 0–4). There were no significant differences between the decitabine‐venetoclax and azacitidine‐venetoclax cohorts with respect to sex, age, race, cytogenetic risk, or comorbidities. Demographics of the decitabine‐venetoclax and the azacitidine‐venetoclax are detailed in Table 1.

**TABLE 1:** Baseline characteristics of patients treated with venetoclax and decitabine or azacitidine.

**Baseline Characteristics**			
**Characteristic**	**All patients** **(*N* = 74)**	**Decitabine‐venetoclax** **(*N* = 48)**	**Azacitidine‐venetoclax** **(*N* = 26)**
Male sex—no. (%)	39 (52.7)	27 (56.3)	12 (46.2)
Age at diagnosis—year			
Median	73	73	73
Range	26–85	26–84	58–85
Race—no. (%)^A^			
Black	23 (31.9)	16 (34.8)	7 (26.9)
White	47 (65.3)	29 (63.0)	18 (69.2)
Other	1 (1.4)	1 (2.2)	1 (3.8)
ELN 2017 cytogenetic risk group—no. (%)^B^			
Favorable	14 (19.7)	9 (19.5)	5 (20.0)
Intermediate	15 (21.1)	9 (19.5)	6 (24.0)
Adverse	42 (59.2)	28 (60.1)	14 (56.0)
Molecular aberrations—no. (%)^C^			
*ASXL1*	15 (23.0)	9 (21.4)	6 (26.1)
*CEBPA* ^biallelic^	2 (3.1)	2 (4.8)	0 (0)
*CEBPA* ^monoallelic^	4 (6.2)	4 (9.5)	0 (0)
*DNMT3A*	12 (18.5)	8 (19.0)	4 (17.4)
*FLT3‐*ITD or *FLT3‐*TKD^D^	18 (27.3)	12 (27.9)	5 (20.8)
*IDH1*	6 (9.2)	2 (4.8)	4 (17.4)
*IDH2*	10 (15.4)	6 (14.3)	4 (17.4)
*KRAS*	6 (9.2)	2 (4.8)	4 (17.4)
*NPM1* ^E^	18 (27.3)	9 (21.4)	9 (37.5)
*NRAS*	13 (20.0)	6 (14.3)	7 (30.4)
*RUNX1*	10 (15.4)	7 (16.7)	3 (13.0)
*SF3B1*	2 (3.1)	0 (0)	2 (8.7)
*SRSF2*	14 (21.5)	8 (19.0)	6 (26.1)
*STAG2*	8 (12.3)	7 (16.7)	1 (4.3)
*TP53*	14 (21.5)	10 (23.8)	4 (17.4)
*U2AF1*	4 (5.4)	3 (7.1)	1 (4.3)
*ZRSR2*	1 (1.5)	1 (2.4)	0 (0)
AML‐MRC—no. (%)^F^	27 (38.6)	20 (42.6)	7 (30.4)
Previously diagnosed MDS—no. (%)	12 (16.2)	7 (14.6)	5 (19.2)
Charlson Comorbidity Index Score			
Median	6	6	6
Range	3–12	4–12	3–12
ECOG at diagnosis			
Median	2	2	2
Range	0–4	0–4	0–4
Stem cell transplant—no. (%)	1 (1.4)	1 (2.1)	0 (0)
Total number of cycles			
Median	2	3	1
Range	1–29	1–29	1–19

A: Race was known in 72 of 74 patients.

B: ELN cytogenetic risk was known in 71 of 74 patients at diagnosis.

C: Sixty‐five of 74 patients had NGS evaluable at diagnosis.

D. One patient was positive for *FLT3*‐ITD by PCR, but no NGS assay was available at diagnosis.

E. One patient was positive for *NPM1* by PCR, but no NGS assay was available at diagnosis.

F: Seventy of 74 patients were evaluable for AML‐MRC at the time of diagnosis.

Molecular profiles were captured at diagnosis on 65 of 74 patients through NGS. The most common mutations were *FLT3*‐ITD or *FLT3‐*TKD (27.3%), *NPM1* (27.3%), *ASXL1* (23.0%), and *TP53* (21.5%). Comprehensive mutational frequencies are detailed in Table 1. Diagnostic bone marrow biopsies were consistent with AML with myelodysplasia‐related changes (AML‐MRC) in 27 (38.6%) of 70 evaluable patients. Twelve (16.2%) were previously diagnosed with a myelodysplastic neoplasm (MDS).

The overall cohort underwent a median of 2 (range, 1–29) cycles of therapy. Patients in the decitabine‐venetoclax cohort underwent a median of 3 (range, 1–29) treatment cycles, and those in the azacitidine‐venetoclax received a median of 1 (range, 1–19) cycle (*p* = 0.077). Only one (2.1%) patient in the overall cohort proceeded to an allogeneic SCT.

### 3.2 Toxicity

Seventy‐one of 74 (95.9%) evaluable patients experienced at least one toxicity of any grade. Hematologic toxicities were the most common grade three or higher adverse events. Significantly fewer patients in the decitabine cohort had lymphocytopenia than the azacitidine group (57.4% vs. 87.5%, respectively, *p* = 0.015). However, significantly more patients in the decitabine cohort had high‐grade thrombocytopenia compared to the azacitidine group (97.9% versus 79.2%, respectively, *p* = 0.015).

The most common grade three or higher non‐hematologic toxicities in the overall cohort were neutropenic fever (43.7%) and infection (40.8%). The most common bacterial infection was *Enterococcus* (17.2%), and the most common viral infection was severe acute respiratory syndrome coronavirus 2 (17.2%). Respiratory failure (16.9%), arrhythmia (7.0%), and hemorrhage (7.0%) were the subsequent most common grade three or higher adverse events. There were no significant differences in high‐grade non‐hematologic toxicities between the decitabine and azacitidine cohorts, presented in Table 2.

**TABLE 2:** Grade three or higher toxicities in patients treated with venetoclax and decitabine or azacitidine.

**Toxicity**				
**Toxicity type**	**All patients** **(*N* = 71)^A^**	**Decitabine‐venetoclax** **(*N* = 47)^B^**	**Azacitidine‐venetoclax** **(*N* = 24)^C^**	**Significance**
Hematologic toxicities, grade ≥3 – no. (%)				
Leukopenia	64 (90.1)	43 (91.5)	21 (87.5)	p = 0.681
Neutropenia	68 (95.8)	46 (97.9)	22 (91.7)	*p* = 0.262
Lymphocytopenia	48 (67.6)	27 (57.4)	21 (87.5)	*p* = 0.015
Anemia	67 (94.4)	46 (97.9)	21 (87.5)	*p* = 0.109
Thrombocytopenia	65 (91.5)	46 (97.9)	19 (79.2)	*p* = 0.015
Non‐hematologic toxicities, grade ≥3 – no. (%)				
Neutropenic fever	31 (43.7)	22 (46.8)	9 (37.5)	*p* = 0.614
Infection	29 (40.8)	21 (44.7)	8 (33.3)	*p* = 0.447
Respiratory failure	12 (16.9)	8 (17.0)	4 (16.7)	*p* > 0.999
Arrhythmia	5 (7.0)	3 (6.4)	2 (8.3)	*p* > 0.999
Hemorrhage	5 (7.0)	4 (8.5)	1 (4.2)	*p* = 0.656
Hypotension	4 (5.6)	3 (6.4)	1 (4.2)	*p* > 0.999
AST elevation	4 (5.6)	2 (4.3)	2 (8.3)	*p* = 0.600
Bilirubin elevation	4 (5.6)	1 (2.1)	3 (12.5)	*p* = 0.109
Creatinine elevation	4 (5.6)	2 (4.3)	2 (8.3)	*p* = 0.600
DIC	3 (4.2)	2 (4.3)	1 (4.2)	*p* > 0.999
Spontaneous TLS	3 (4.2)	2 (4.3)	1 (4.2)	*p* > 0.999
ALT elevation	2 (2.8)	1 (2.1)	1 (4.2)	*p* > 0.999
Therapy‐related TLS	1 (1.4)	1 (2.1)	0 (0)	*p* > 0.999
Death during induction—no. (%)^D^				
Death within 30 days	7 (9.6)	4 (8.3)	3 (12.0)	*p* = 0.685
Death within 60 days	12 (16.4)	5 (10.4)	7 (28.0)	*p* = 0.093

A: Seventy‐one of 74 evaluable patients had at least one toxicity.

B: Forty‐seven of 48 evaluable patients in the decitabine cohort had at least one toxicity.

C: Twenty‐four of 26 evaluable patients in the azacitidine cohort had at least one toxicity.

D: All 48 patients in the decitabine cohort were evaluable for mortality during induction. Death during induction was evaluable in 25 of 26 patients in the azacitidine cohort.

**FIGURE 2:**
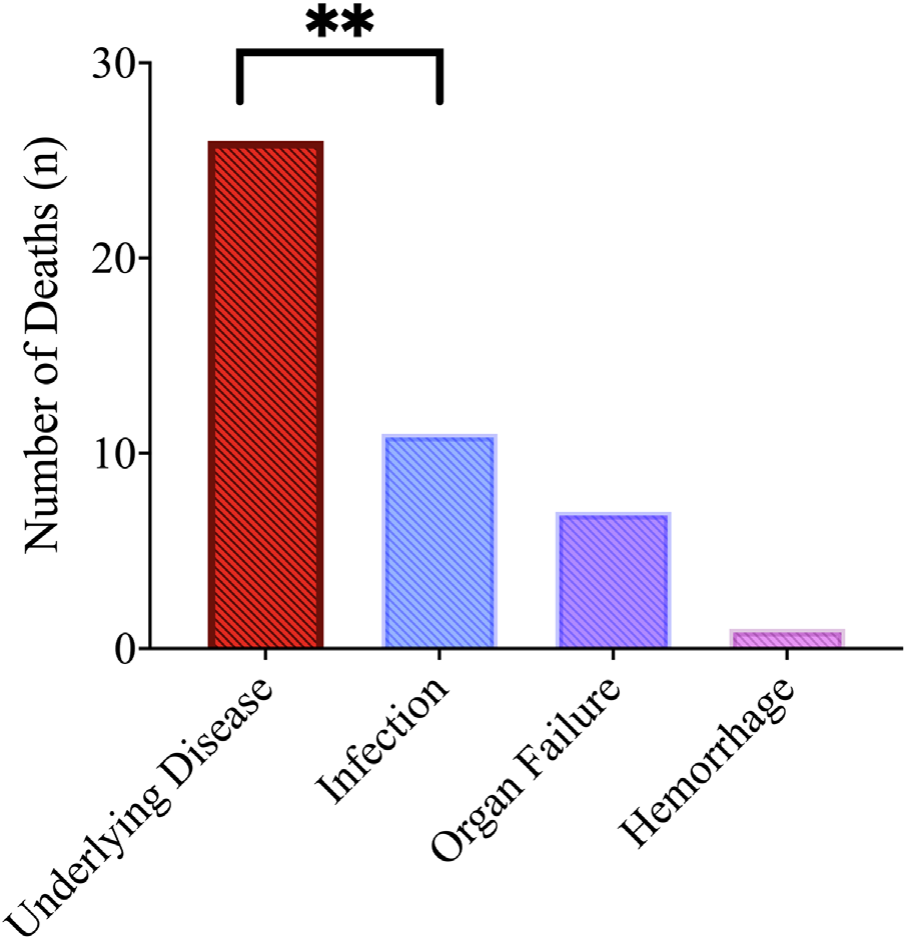
Causes of death of patients treated with venetoclax and decitabine or azacitidine. ** *p* = 0.002.

Death during treatment was evaluable in 73 of 74 patients. Death within 30 days occurred in 9.6%; 16.4% died within 60 days. Deaths within 30 and 60 days were non‐significantly higher in the azacitidine‐venetoclax cohort compared to decitabine‐venetoclax, with more deaths within 60 days in the azacitidine‐venetoclax cohort approaching significance (*p* = 0.093). The cause of death was known in 42 patients. Death from relapsed or refractory disease (61.9%) was significantly higher than death from infection (26.2%, *p* = 0.002), organ failure (16.7%, *p* = 0.0001), or hemorrhage (2.4%, *p* = 0.0001), depicted in Figure 2.

### 3.3 Response

Sixty‐six of 74 patients were evaluable for response in the overall cohort. Three (4.5%) patients achieved CR, 18 (27.3%) achieved CRi, and five (7.8%) achieved CRh. The composite complete remission (CRc) rate was 39.4% (95% confidence interval [CI], 28.5–51.5). In the decitabine‐venetoclax cohort, the CRc rate was 44.2% (95% CI, 30.4–58.9) compared to 30.4% (95% CI, 15.6–50.9) in the azacitidine‐venetoclax cohort. There were no significant differences in remission rates between the decitabine and azacitidine cohorts (*p* = 0.304).

Next, we investigated how the ELN 2017 risk classification impacts response between decitabine‐venetoclax and azacitidine‐venetoclax. The CRc rates were 41.2% for favorable risk, 38.5% for intermediate risk, and 44.4% for adverse cytogenetic risk. There were no significant differences in response rates across cytogenetic risk categories.

We identified 31 patients with AML‐MRC or a diagnosis of MDS prior to AML, and 28 were evaluable for a response: the CRc rate was 39.3% (95% CI, 23.6–57.6). By comparison, 22 evaluable patients with *de novo* AML had a CRc rate of 36.4% (95% CI, 19.7–57.0), and no significant difference was observed between the two cohorts (*p* > 0.999).

**FIGURE 3:**
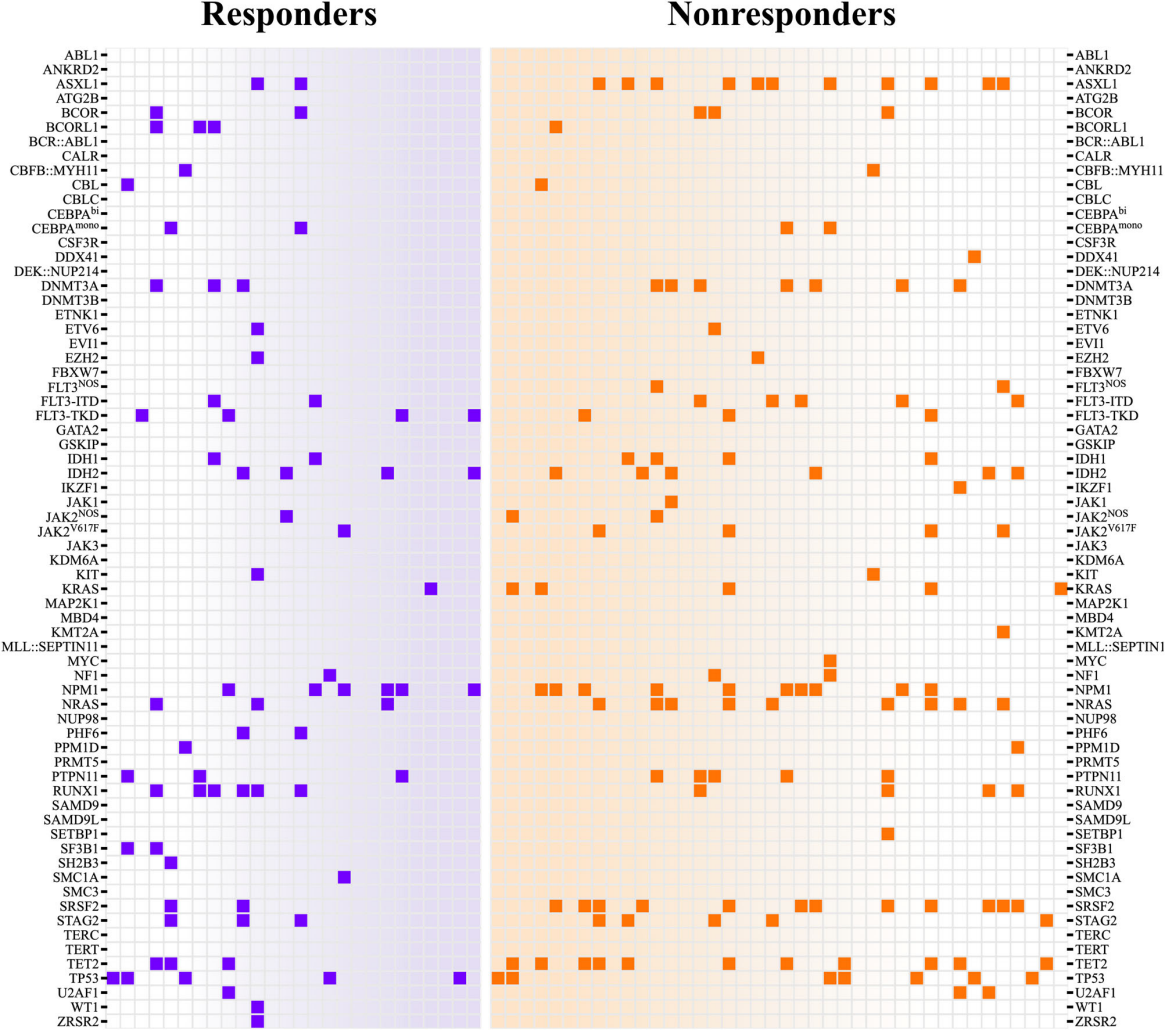
Heat map of mutations in venetoclax‐sensitive and venetoclax‐resistant patients. Patients that achieved complete remission (CR), CR with incomplete hematologic recovery (CRi), or CR with partial hematologic recovery (CRh) are shown in the left panel. Patients that were refractory to venetoclax, died during treatment, or had an evaluable response that was no better than a morphological leukemia‐free state are shown in the right panel.

Eight patients received a prior hypomethylating agent for preceding MDS or chronic myelomonocytic leukemia (CMML). The CRc rate was 28.6% when therapy was escalated to venetoclax and a hypomethylating agent, compared to 40.7% in patients without prior hypomethylating agent exposure (*p* = 0.695). The response rates are detailed in Table 3, and a heat map of molecular profiles among responders and non‐responders is depicted in Figure 3.

**TABLE 3:** Response of patients treated with venetoclax and decitabine or azacitidine.

**Response**				
**Response Category**	**All patients** **(*N* = 66)^A^**	**Decitabine‐venetoclax** **(*N* = 43)^B^**	**Azacitidine‐venetoclax** **(*N* = 23)^C^**	**Significance**
Complete remission (CR)	3 (4.5)	2 (4.7)	1 (4.3)	*p* > 0.999
Complete remission with incomplete hematologic recovery (CRi)	18 (27.3)	14 (32.6)	4 (17.4)	*p* = 0.251
Complete remission with partial hematologic recovery (CRh)	5 (7.8)	3 (7.0)	2 (8.7)	*p* > 0.999
Composite complete remission (CR + CRi + CRh)	26 (39.4)	19 (44.2)	7 (30.4)	*p* = 0.304
Composite complete remission by ELN 2017 cytogenetic risk category—no. (%)^D^				
Favorable^E^	5 (41.2)	3 (42.9)	2 (40.0)	*p* > 0.999
Intermediate^F^	5 (38.5)	4 (50.0)	1 (20.0)	*p* = 0.301
Adverse^G^	16 (44.4)	12 (52.2)	4 (30.7)	*p* = 0.301

A: Sixty‐six of 74 patients were evaluable for response.

B: Forty‐three of 48 evaluable patients in the decitabine cohort were evaluable for response.

C: Twenty‐three of 26 evaluable patients in the azacitidine cohort were evaluable for response.

D: Forty‐six of 48 patients in the decitabine cohort and 25 of 26 in the azacitidine cohort had ELN 2017 risk known at diagnosis.

E: Seven of nine favorable‐risk patients in the decitabine cohort were evaluable for response.

F: Eight of nine intermediate‐risk patients in the decitabine cohort and five of six intermediate‐risk patients in the azacitidine cohort were evaluable for response.

G: Twenty‐three of 26 adverse‐risk patients in the decitabine cohort and 13 of 14 adverse‐risk patients in the azacitidine cohort were evaluable for response.

### 3.4 Survival

**FIGURE 4:**
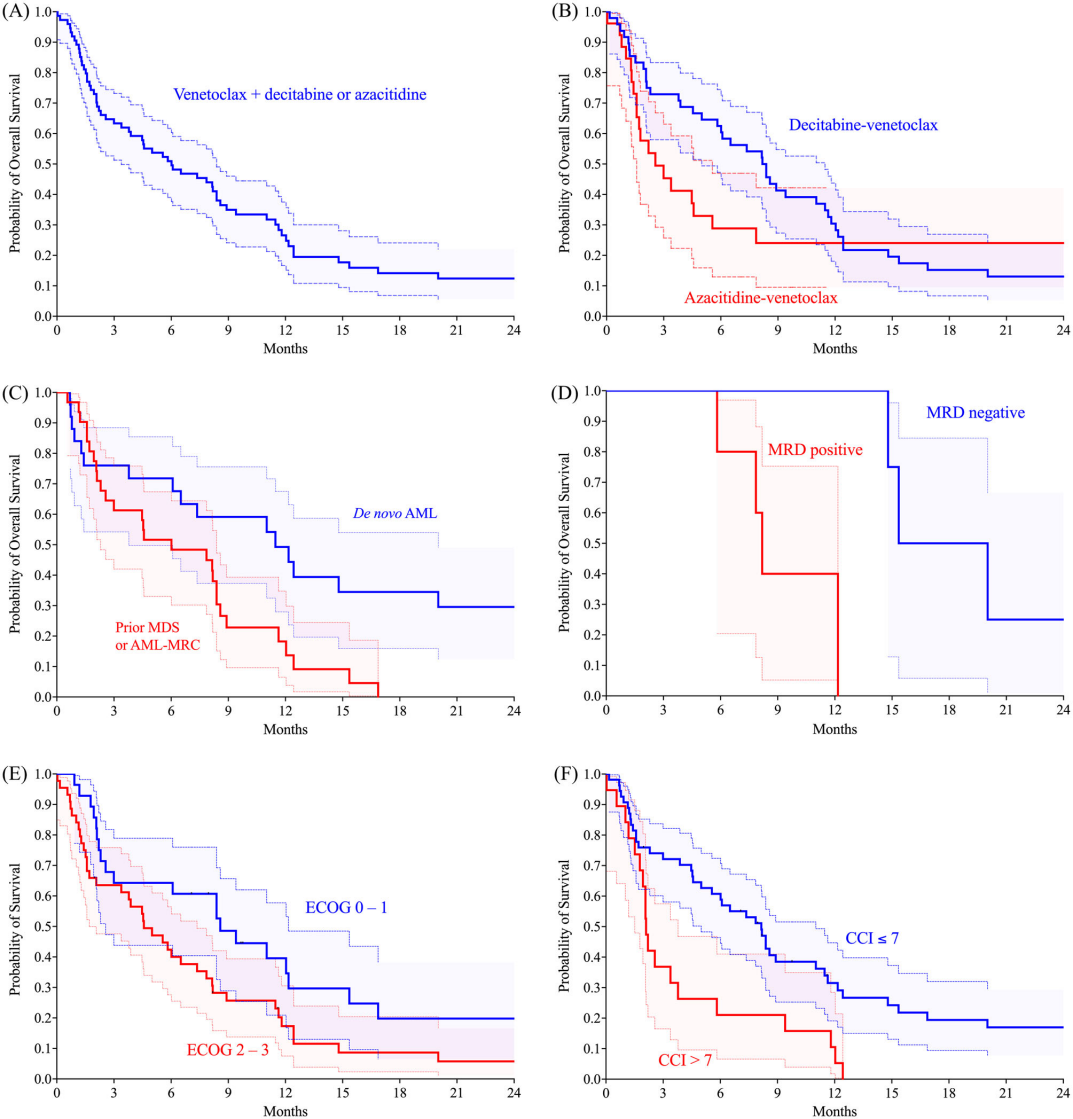
(A) Overall survival of venetoclax and decitabine or azacitidine was 6.0 months. (B) Median overall survival of decitabine‐venetoclax was 8.3 months versus 2.6 months with venetoclax (*p* = 0.080). (C) Median overall survival of the *de novo* acute myeloid leukemia (AML) cohort was 11.5 months versus 6.0 months in the MDS/AML‐MRC group (*p* = 0.01). D. Median overall survival was 17.7 months for measurable residual disease (MRD) negative versus 8.2 months for MRD positive (*p* = 0.01). E. Median overall survival was 8.6 months for Eastern Cooperative Oncology Group (ECOG) 0–1 versus 4.6 months for ECOG 2–3 (*p* = 0.034). F. Median overall survival of CCI score ≤7 (8.2 months) versus > 7 (2.1 months) (*p* = 0.002).

The median overall survival for the entire cohort was 6.0 months, and the median duration of follow‐up was 33.0 months, shown in Figure 4A. In the decitabine‐venetoclax cohort, the median overall survival was 8.3 months compared to 2.6 months in the azacitidine‐venetoclax group, approaching statistical significance (*p* = 0.080, Figure 4B). The overall survival data are summarized in Table 4.

**TABLE 4:** Survival of patients treated with venetoclax and decitabine or azacitidine

**Survival**				
	**All patients** **(*N* = 74)**	**Decitabine‐venetoclax** **(*N* = 48)**	**Azacitidine‐venetoclax** **(*N* = 26)**	**Significance**
Median overall survival—m.	6.0 m	8.3 m	2.6 m	*p* = 0.080
Progression‐free survival—m.	4.5 m	6.1 m	2.6 m	*p* = 0.129
Overall survival by ELN 2017 cytogenetic risk category—no. (%)				
Favorable	7.6 m	12.2 m	1.7 m	*p* = 0.274
Intermediate	4.5 m	5.8 m	2.1 m	*p* = 0.031
Adverse	8.2 m	8.4 m	4.0 m	*p* = 0.439

In the entire cohort, the median overall survival was 7.6 months in the ELN 2017 favorable risk group, 4.5 months for intermediate risk, and 8.2 months for adverse risk, with no significant differences between cytogenetic risk cohorts. Within the intermediate‐risk category, survival significantly favored the decitabine‐venetoclax group at 5.8 months compared to the azacitidine‐venetoclax group at 2.1 months (*p* = 0.031).

Next, we compared patients with a diagnosis of MDS preceding AML or AML‐MRC with *de novo* AML. The median overall survival significantly favored the *de novo* cohort at 11.5 months compared to 6.0 months in the preceding MDS/AML‐MRC group (*p* = 0.01, Figure 4C). Patients exposed to a prior hypomethylating agent for preceding MDS or CMML had a median overall survival of 6.8 months compared to 6.0 months for those without previous hypomethylating agent exposure (*p* = 0.602).

Subgroup analyses were performed and stratified by molecular cohort. *IDH1* ^mut^ and *IDH2* ^mut^ AML was associated with significantly prolonged survival by Cox proportional hazards regression compared to *IDH* wild‐type (hazard ratio for death, 0.26; 95% CI, 0.09–0.61, *p* = 0.005). Patients with *NRAS* ^mut^ and *KRAS* ^mut^ were associated with reduced survival compared to patients without signaling mutations in *RAS*, *FLT3*, or *KIT*, approaching statistical significance (hazard ratio for death, 2.0; 95% CI, 0.96–3.84, *p* = 0.057).

Patients with an ECOG score of 0 – 1 had significantly prolonged survival at 8.6 months compared to patients with an ECOG score of 2 – 3 at 4.6 months (*p* = 0.034, Figure 4E). Patients with CCI scores <7 had a median overall survival of 8.2 months compared to 2.1 months for scores ≥7 (*p* = 0.002, Figure 4F). Therefore, we identified a CCI score threshold of ≥7, which distinguished a cohort of patients with a significantly higher risk of death.

### 3.5 MRD status

Patients that achieved CR, CRi, and CRh were analyzed for survival with respect to MRD positivity. Patients that responded and were MRD negative had a significantly prolonged median overall survival of 17.7 months compared to 8.2 months for patients that were MRD positive (*p* = 0.01, Figure 4D).

## 4 DISCUSSION

We highlight several novel observations in the real‐world setting, in addition to evidence supporting clinical trial findings for toxicity, response, and survival. We demonstrate that decitabine‐venetoclax may result in significantly more severe thrombocytopenia than azacitidine‐venetoclax, which may extend periods of transfusion dependence and cycle delays for count recovery. In contrast, the degree of severe lymphocytopenia was significantly lower in the decitabine‐venetoclax cohort — implying more profound cytopenias may not universally be associated with decitabine‐venetoclax across all cell lines. The limitations of toxicity analysis stem from the retrospective nature of this study. While quantitative toxicity grading was well‐detailed, qualitative toxicity grading should be interpreted with caution, given the limitations of retrospective studies.

We noted a potentially concerning and non‐significantly elevated proportion of death within 60 days in the azacitidine‐venetoclax cohort at 40.0% compared to 18.7% in the decitabine‐venetoclax group. However, we emphasize that during the cause‐of‐death analysis, significantly more patients died of relapsed or refractory disease rather than infection, organ failure, hemorrhage, or other toxicities. Given these findings, strategies to augment venetoclax response, reduce venetoclax resistance, and identify cohorts that preferentially respond to venetoclax‐based combinations should be investigated.

We observed a composite complete remission rate of 39.4% in the overall cohort, with similar response rates between ELN 2017 cytogenetic risk categories. The favorable and intermediate cytogenetic risk categories exhibited substantially shorter overall survival than expected, with the survival of both categories being non‐significantly shorter than the adverse category. An explanation for these findings may be due to patterns of molecular segregation across cytogenetic risk categories, implicating venetoclax resistance. For example, *RAS* mutations are more likely to be associated with favorable and intermediate karyotypes [26]. Our findings suggest that the ELN 2017 risk categorization is not optimized for lower‐intensity venetoclax‐based strategies.

Our subset analyses identified that *IDH* ^mut^ AML had significantly improved survival compared to *IDH* wild‐type, suggesting venetoclax may be beneficial in specific molecular cohorts [27]. In contrast, signaling mutations, such as *NRAS* ^mut^ or *KRAS* ^mut^, appeared to be associated with reduced survival. Our results suggest that a revised risk stratification schema incorporating *IDH* and *RAS* mutational status may more accurately predict the prognosis of patients treated with a hypomethylating agent and venetoclax. Furthermore, patients with an MRD‐negative response at any time had significantly prolonged overall survival than those with an MRD‐positive response. Therefore, the incorporation of MRD status may further refine a revised risk stratification model.

The median overall survival was 6.0 months in the entire cohort, lower than observed in VIALE‐A at 14.7 months. We hypothesize that there are several explanations for this finding. In the azacitidine‐venetoclax cohort, the median number of cycles was one, and in the overall group, the median number of cycles was two — a stark contrast to a median of seven cycles in VIALE‐A. Fewer cycles administered and higher rates of early death likely contribute to shorter survival outside of clinical trials.

Due to the decreased survival outside of clinical trials, the selection of therapy candidates needs to be improved. We demonstrated that ECOG scores of 0 to 1 were associated with significantly improved survival compared to ECOG scores of 2 to 3. More strikingly, we identified that a CCI score threshold of seven differentiated patients is more likely to have favorable outcomes despite numerous comorbidities. This threshold may guide candidates for venetoclax‐based therapy and reduce the rates of treatment‐associated mortality and early death.

Overall, the combination of venetoclax and a hypomethylating agent is associated with lower remission rates and overall survival outside clinical trials. We emphasize three important conclusions of this study: toxicities do not appear to be uniform between decitabine and azacitidine backbones, the ELN 2017 risk stratification is not optimized for patients treated with venetoclax and a hypomethylating agent, and the CCI score refines the selection of treatment candidates outside of clinical trials.

## AUTHOR CONTRIBUTIONS

Ian M. Bouligny analyzed the data, created the figures, and prepared the manuscript. Graeme Murray collected the data and revised the manuscript. Michael Doyel collected the data and revised the manuscript. Tilak Patel collected the data and revised the manuscript. Josh Boron collected the data and revised the manuscript. Valerie Tran assisted with the REDCap instrument development and revised the manuscript. Juhi Gor assisted with the REDCap instrument development and revised the manuscript. Yiwei Hang assisted with the REDCap instrument development and revised the manuscript. Yanal Alnimer assisted with the REDCap instrument development and revised the manuscript. Kyle Zacholski assisted with the REDCap instrument development and revised the manuscript. Chad Venn assisted with the REDCap instrument development and revised the manuscript. Nolan A. Wages analyzed the data and revised the manuscript. Steven Grant oversaw the project and revised the manuscript. Keri R. Maher oversaw the project and revised the manuscript.

## CONFLICT OF INTEREST STATEMENT

The authors declare no conflict of interest.

## PATIENT CONSENT STATEMENT

Patient consent was waived by the Institutional Review Board of Virginia Commonwealth University Medical Center.

## Data Availability

The data that support the findings of this study are available from the corresponding author upon reasonable request.
